# Self-management advice, exercise and foot orthoses for plantar heel pain: the TREADON pilot and feasibility randomised trial

**DOI:** 10.1186/s40814-021-00808-0

**Published:** 2021-04-01

**Authors:** Linda S. Chesterton, Martin J. Thomas, Gordon Hendry, Ying Chen, David Goddin, Nicola Halliday, Sarah A. Lawton, Martyn Lewis, Christian D. Mallen, Hylton B. Menz, Nadine E. Foster, Edward Roddy

**Affiliations:** 1 Primary Care Centre Versus Arthritis, School of Medicine, Keele University, Keele, Staffordshire ST5 5BG UK; 2 Haywood Academic Rheumatology Centre, Midlands Partnership NHS Foundation Trust, Burslem, Staffordshire ST6 7AG UK; 3 School of Health and Life Sciences, Glasgow Caledonian University, Glasgow, G4 0BA UK; 4 Keele Clinical Trials Unit, David Weatherall Building, Keele University, Keele, Staffordshire ST5 5BG UK; 5 Department of Health and Environmental Sciences, Xi’an Jiaotong-Liverpool University, Suzhou, 215123 China; 6 Discipline of Podiatry and La Trobe Sport and Exercise Medicine Research Centre, School of Allied Health, Human Services and Sport, La Trobe University, Bundoora, VIC 3086 Australia

**Keywords:** Randomised trial, Pilot and feasibility trial, Plantar heel pain/fasciitis, Foot orthoses, Exercise

## Abstract

**Background:**

Plantar heel pain (PHP) is common and impacts negatively on physical function and quality of life. Initial treatment usually comprises analgesia and self-management advice (SMA), with referral to a physiotherapist or podiatrist recommended only when symptoms persist. Systematic reviews highlight limitations of existing evidence for the effectiveness of exercises and orthoses. The objective of the TREADON pilot and feasibility trial was to inform the design of a future main trial to compare the clinical and cost-effectiveness of self-management advice (SMA), individualised exercises and foot orthoses for PHP.

**Methods:**

This was a four-arm randomised feasibility and pilot trial with 12-week follow-up. Adults aged ≥ 18 years with PHP were identified from primary care by general practice consultation, retrospective general practice medical record review or a population survey. Participants were randomised to either (i) SMA, (ii) SMA plus individualised exercises (SMA-exercises), (iii) SMA plus prefabricated foot orthoses (SMA-orthoses) or (iv) SMA plus combined individualised exercises and prefabricated foot orthoses (SMA-combined). Feasibility outcomes were recruitment; retention; intervention adherence, credibility and satisfaction; performance of three potential primary outcome measures (pain numeric rating scale (NRS), Foot Function Index-pain subscale (FFI-pain), Manchester Foot Pain and Disability Index-pain subscale (MFPDI-pain)); and parameters for informing the main trial sample size calculation.

**Results:**

Eighty-two participants were recruited. All three identification methods met the target number of participants. Retention at 12 weeks was 67%. All interventions were successfully delivered as per protocol. Adherence (range over 12 weeks 64–100%) and credibility (93%) were highest in the SMA-combined arm. Satisfaction with treatment was higher for the three clinician-supported interventions (SMA 29%, SMA-exercises 72%, SMA-orthoses 71%, SMA-combined 73%). Responsiveness (baseline to 12 weeks) was higher for FFI-pain (standardised response mean 0.96) and pain NRS (1.04) than MFPDI-pain (0.57). Conservative sample size parameter estimates for standard deviation were pain NRS 2.5, FFI-pain 25 and MFPDI-pain 4, and baseline-outcome correlations were 0.5–0.6, 0.4 and < 0.3, respectively.

**Conclusions:**

We demonstrated the feasibility of conducting a future main randomised clinical trial comparing the clinical and cost-effectiveness of SMA, exercises and/or foot orthoses for PHP.

**Trial registration number:**

ISRCTN 12160508. Prospectively registered 5^th^ July 2016.

**Supplementary Information:**

The online version contains supplementary material available at 10.1186/s40814-021-00808-0.

## Key messages regarding feasibility

1. What uncertainties existed regarding the feasibility?

To inform the design of a future main randomised trial of self-management advice, exercise and orthoses for plantar heel pain, we compared the success of three different participant identification methods, determined the number of trial arms and which interventions to test, compared the performance of three potential primary outcome measures and investigated key parameters to inform the sample size calculation. We investigated whether physiotherapists and podiatrists were able to deliver the interventions to protocol in a similar way.

2) What are the key feasibility findings?

All three identification methods met the target number of participants. Loss-to-follow-up rates were acceptable but would need to be improved in a future main trial. The interventions achieved high levels of adherence, satisfaction and credibility and were delivered successfully by both physiotherapists and podiatrists. The three potential primary outcome measures were comparable, although the Manchester Foot Pain and Disability Index had lower responsiveness. Key information to determine the sample size calculation was obtained.

3) What are the implications of the feasibility findings for the design of the main study?

A future main randomised clinical trial testing these interventions is feasible and acceptable. Either the pain numeric rating scale or the Foot Function Index would be suitable as the primary outcome measure for the future main trial.

## Background

Plantar heel pain (PHP) describes a range of conditions affecting the plantar heel of unknown aetiology [1]. It is typically aggravated by weight-bearing after prolonged rest. PHP affects 10% of adults during their lifetime, impairing quality of life, physical function, mobility and ability to work [2–4]. PHP is usually self-limiting, although symptoms can take 1 to 2 years to resolve [5]. Primary care treatment approaches comprise analgesia and self-management advice regarding rest, footwear, heel pads and weight loss [5–7]. Referral to a physiotherapist or podiatrist is recommended if symptoms persist [5, 8]. However, symptoms can become chronic and persistent, leading to impaired quality of life, physical inactivity and weight gain [4, 9].

Physiotherapists and podiatrists typically use lower limb exercises and/or foot orthoses to treat PHP [10–15], although the evidence supporting these interventions is limited. Systematic reviews highlight that many trials are limited by small sample sizes, poor methodological quality and short duration of follow-up [16–18]. Recent reviews of foot orthoses reached conflicting conclusions about their effectiveness relative to sham orthoses [16, 17], and our recent network meta-analysis found limited evidence about which conservative treatment is the most effective for PHP [18]. Hence, a large, methodologically robust multicentre randomised trial that compares exercises and orthoses for the management of PHP over a longer follow-up period is needed. We undertook the TREADON (TReatments of Exercise AnD Orthotic devices for plaNtar heel pain**)** feasibility and pilot trial to (i) compare the success of three different identification methods, (ii) determine the number of trial arms and which interventions to test, (iii) determine the primary outcome measure, (iv) inform the sample size calculation and (v) examine potential prognostic baseline factors.

## Methods

### Design

This was a pragmatic, four-parallel-arm, multicentre, randomised, feasibility and pilot trial undertaken in 12 general practices and 2 NHS trusts. The trial protocol is publically available [19].

### Participants

Participants were community-dwelling adults aged ≥ 18 years identified from participating general practices. Inclusion criteria were self-reported localised pain under the heel aggravated by weight-bearing, worst when first standing or after rest, especially on getting up in the morning or following periods of prolonged sitting; symptom duration of at least 4 weeks; minimum pain score of 2 on a 0–10 numeric rating scale (NRS); owning/access to a mobile or landline telephone; and being able and willing to participate and provide written informed consent. Exclusion criteria were inflammatory arthritis (e.g. rheumatoid arthritis, ankylosing spondylitis, reactive arthritis, systemic lupus erythematosus, gout, psoriatic arthritis); fibromyalgia; serious pathologies (e.g. malignancy, trauma, infection); treatment for PHP by a physiotherapist or podiatrist currently or in the last 3 months; corticosteroid injection into the affected foot in the last 3 months; previous or awaiting surgery on the affected foot; allergy to common orthotic device materials (e.g. adhesives, latex, sock dyes, certain shoe types); or unwillingness/inability to undertake interventions or attend clinics, complete follow-up questionnaires in English or receive text messages/phone calls.

### Participant identification and recruitment

Potential participants were identified by three methods:

(i) General practice consultation (nine general practices): general practitioners (GP) were alerted by electronic ‘pop-up’ reminders when they entered a relevant PHP diagnostic code. Patients were screened for potential eligibility by the GP. Potentially eligible, interested patients were given a participant information leaflet (PIL) and asked for written consent to be contacted by the research team. GPs provided advice and, if appropriate, pain relief medication but no other interventions. Patients were posted a trial information pack (invitation letter, PIL, consent form, baseline questionnaire and pre-paid return envelope).
(ii) Retrospective medical record review (MRR) (11 general practices): patients who had consulted their GP for foot/ankle pain in the preceding year were identified from medical records. A broad range of symptom codes was used because foot/ankle consultations are often not coded with specific diagnostic labels such as PHP [20]. GPs screened lists to exclude potentially vulnerable patients. Remaining patients were mailed a brief information leaflet, screening survey, consent to contact form and pre-paid return envelope. The survey asked respondents to indicate the location of pain on a validated foot manikin (© The University of Manchester 2000. All rights reserved) [21, 22] and complete questions regarding foot pain, demographic details and eligibility. Non-responders were sent reminders after 2 and 4 weeks.
(iii) Population survey (one general practice): a postal questionnaire was mailed to all adults registered at one general practice to identify those who had heel pain but had not consulted in the preceding year. GPs screened lists to exclude potentially vulnerable patients. Patients were mailed as described in (ii).

Patients identified via (ii) and (iii) who provided consent to contact and who appeared eligible were then posted a trial information pack.

Patients sent a trial information pack by any of the three methods were telephoned to explain the trial, confirm eligibility and obtain consent to participate. The completed consent form and baseline questionnaire were returned by postal mail.

### Randomisation and concealment

On receipt of the completed consent form and baseline questionnaire, participants were randomly allocated to an intervention by administrators using the Keele Clinical Trials Unit secure randomisation system. Allocation was concealed from the researchers. Stratified block randomisation was undertaken (fixed block size 4), blocked by treatment site. Participants were randomised on an equal basis to one of the following interventions:

(i) Self-management advice (SMA) booklet: control arm
(ii) SMA booklet plus individualised exercise (SMA-exercises)
(iii) SMA booklet plus pre-fabricated foot orthoses (SMA-orthoses)
(iv) SMA booklet plus individualised exercise and pre-fabricated foot orthoses (SMA-combined)

Owing to the nature of the interventions, participants could not be blind to treatment allocation. Due to slow recruitment initially, and with agreement from the Trial Steering Committee and funder, randomisation to the SMA control arm ceased following allocation of 11 participants to this arm. Subsequent participants were randomised on a 1:1:1 basis to the three clinician-supported intervention arms to ensure sufficient data were collected to assess the feasibility of these interventions. Participants were informed of their allocation in writing.

### Interventions

#### SMA control arm

Participants were posted a bespoke SMA booklet about PHP, which included stretching exercises reproduced with permission from the Versus Arthritis PHP exercise sheet and supplemented with specific advice and information including self-help messages about pain relief, footwear, rest and weight loss [23].

#### SMA-exercises

Participants were given the SMA booklet. The treating clinician (a physiotherapist or podiatrist) assessed point tenderness under the heel by pressing the thumb into the underside of the heel (medial calcaneal tubercle) and medial longitudinal arch, and documented foot posture using the Foot Posture Index-6 (FPI-6) [24]. They could receive an additional generic lower limb assessment of alignment and function if deemed appropriate. Exercise selection was informed by the degree of clinically observed muscle tightness, weakness and functional limitation. The exercises were drawn from best available evidence [10, 25–29] and discussion with local clinicians during a pre-trial workshop, and included foot-specific stretches/exercises targeting the plantar fascia and intrinsic foot muscles, ankle-related muscle groups such as soleus and gastrocnemius and other lower limb muscle groups (Additional file 1). Participants were instructed how to perform the exercises, advised on dose (frequency, intensity, type and timing) and given an individualised and detailed exercise sheet to support their home exercise programme. The individualised exercise programme was progressed at follow-up consultations informed by subjective and objective re-assessment.

#### SMA-foot orthoses

Participants were given the SMA booklet and were assessed as described above. The orthotic device was chosen based on the degree of static rearfoot eversion assessed by the FPI-6 (Additional file 2). Participants were instructed how to fit the device and advised to wear it for 1 h per day, gradually increasing by 1 h per day up to at least 4 h per day, and given an individualised and detailed orthosis information sheet. The orthosis could be altered during subsequent consultations according to participants’ self-reported tolerance or clinical presentation.

#### SMA-combined

Participants were given the SMA booklet, underwent clinical assessment and received both exercise and orthoses interventions as described above.

Exercise and orthosis interventions were delivered over up to six treatment sessions over 12 weeks by a NHS physiotherapist or podiatrist trained to deliver all intervention protocols. Clinicians attended a 2-day training workshop prior to the start of recruitment and treatment, covering carrying out the standardised assessment, delivery of the interventions in line with the agreed protocol and documentation including case report forms and adverse event reporting, supplemented by a comprehensive clinician manual, providing clear treatment protocols.

Participants were asked not to use other treatments during the 12-week intervention period, other than medication if this had been provided by their GP.

### Follow-up and outcomes

#### Data collection

Participants rated their average PHP intensity in the last 7 days (0–10 NRS, anchored 0 = ‘no pain’ and 10 = ‘worst pain imaginable’). Pain NRS were collected weekly for 12 weeks by text message or brief telephone call. Other outcomes were collected by postal questionnaire at 12 weeks. Non-responders to the questionnaire were sent a reminder text message/postcard after 10 days and a second questionnaire after a further five days, and then telephoned by a research nurse to collect key outcomes. Non-responders unable to be contacted by telephone were mailed a brief minimum data questionnaire. Participants randomised to the clinician-supported intervention arms recorded adherence, engagement with the intervention and adverse events in a weekly paper diary.

#### Process outcomes

The feasibility of the patient identification methods was assessed by comparing the number identified, eligible and recruited, rates of recruitment and retention, and by evaluating the cost and effort of each method.

Participant-reported engagement with and adherence to each intervention was assessed including a global intervention adherence scale (5-point Likert scale; doing exercises/wearing orthoses as often as advised over the last week; strongly agree/agree/not sure/disagree/strongly disagree), number of weeks adherence, the duration and frequency of completing exercises and/or wearing foot orthoses and reasons for non-adherence. Participant-reported intervention credibility and satisfaction were assessed. Fidelity of intervention delivery and participant attendance at intervention sessions were collected using clinician-completed case report forms. Participating clinicians’ evaluations of intervention delivery, including acceptability of the FPI-6, views on the number and duration of intervention sessions, ease of generation of individualised exercise regimen sheets and use of the orthotic devices were assessed via a short online survey and facilitated workshop at the end of the trial.

#### Clinical outcomes

Three potential primary outcome measures for the future main trial were evaluated:

(i) PHP 0-10 pain intensity NRS. Further evaluation was undertaken exploring time to participants’ report of being ‘significantly better’ defined as a NRS score of 0 or 1 recorded over two consecutive weeks.
(ii) Change in the Foot Function Index pain subscale (FFI-pain) from baseline to 12 weeks, a validated nine-item self-administered questionnaire [30, 31].
(iii) Change in the Manchester Foot Pain and Disability Index pain subscale (MFPDI-pain) from baseline to 12 weeks, a validated seven-item self-administered questionnaire [32].

Completion rates of secondary outcome measures were assessed. Presence of PHP (yes/no) was assessed at baseline, in weekly text messages and at 12 weeks. First step pain (0–10 NRS), foot function (FFI disability and activity restriction subscales, MFPDI function subscale) [30–32], health-related quality of life (EQ-5D-5L) [33], current employment status and work performance (on average, to what extent has your heel pain affected your performance at work over the past month? 0–10 NRS anchored 0 = ‘not at all’ and 10 = ‘so bad I am unable to do my job’) and absenteeism (number of days lost) were assessed at baseline and 12 weeks. Participant impression of change in heel pain over 12 weeks (six response options: completely recovered, much better, better, no change, worse and much worse) and healthcare utilisation (including use of analgesics) were self-reported by participants at 12 weeks. Adverse events were reported by participants in the weekly diary and 12-week questionnaire and also by GPs and treating physiotherapists and podiatrists.

### Sample size

We aimed to derive sufficiently precise estimates for the future main trial around: (i) overall adherence to intervention protocols (90% one-sided lower confidence bound), (ii) observed completion rates of the FFI and MFPDI (90% one-sided lower confidence bound), and (iii) the standard deviation (SD) for the pain NRS, FFI and MFPDI (at the level of an inflation factor of 1.1 in the point estimate of the feasibility SD) providing 80–90% confidence in attaining the nominal power of the main trial [34]. We estimated that we would need to recruit 80 participants (20 per arm) over a 9-month period.

### Statistical analysis

Results were analysed descriptively and there was no emphasis on hypothesis-testing. At baseline and follow-up, participants’ descriptive data were summarised using median (inter-quartile range (IQR)) or mean (SD), and frequency counts and percentages for categorical variables overall and by intervention arm.

Rates of eligibility and recruitment (with 80%, 90% and 95% confidence intervals (CI), number of consenting/randomised participants per month) and retention (returned 12-week questionnaires, number of weekly texts) were estimated, in total and stratified by identification method. Participants’ baseline characteristics were examined and compared to ineligible and non-consenting patients and between the three identification methods.

Fidelity and adherence to intervention protocols were compared between arms including attendance at intervention sessions, adherence to exercise and orthosis interventions and intervention credibility and satisfaction, summarising categorical variables using counts and percentages. Clinician survey and workshop data were analysed descriptively.

Performance of the three potential primary outcome measures was assessed using response, item completion rates, floor/ceiling effects and responsiveness (standardised response mean) [35]. Analysis of outcome performance comprised (i) mean pain scores (standard deviation) overall and for each week of follow-up and/or 12 week follow-up, (ii) life-table and Kaplan-Meier survival plots with an emphasis on mean survival times for pain NRS time-to-event data, and (iii) the percentage of participants meeting recognised minimal clinically important difference (MCID) thresholds (where available), i.e. medians of 1.7 and 12 for pain NRS and FFI-pain respectively [36, 37].

Effect size, standard deviation, baseline-outcome correlation, inter-correlation of pain NRS scores (for repeated measures) and dropouts to inform the sample size calculation for the main trial were calculated.

Associations between baseline variables (first episode of PHP, bilateral PHP, older age, duration of PHP, baseline pain score, morning pain (0–10 NRS), employment status (employed/not employed), baseline general health (EQ-5D-5L)) and the three clinical pain outcomes were calculated using Spearman’s correlation; coefficients exceeding a pre-agreed cut-off (*r*_s_ ≥ 0.3) were considered suitable for taking forward as potential baseline covariates in regression-based adjustment within the main trial.

The feasibility of collecting healthcare resource use and quality of life data was determined by examining response and item completion rates. Costs were derived by linking intervention costs and healthcare resource use costs [38–40] obtained from self-reported data with available unit costs. Aggregated and disaggregated costs were descriptively compared between intervention arms.

Final analysis used STATA version 14.2 (StataCorp LLC, College Station, TX) and was completed after the final 12-week follow-up.

### Patient and public involvement

This trial was developed with research users with PHP who provided feedback on the funding application and protocol, particularly the content of the exercises and the choice of orthoses. They also advised on the potential primary outcome measures for the future main trial which were evaluated in the pilot and feasibility trial and the content of participant-facing paperwork and text messages. The information and advice provided in the SMA booklet was developed with input from four people with PHP and members of Keele’s Patient and Public Involvement and Engagement group over three 1–2 h workshops. One patient representative served on the independent trial steering committee, playing a full part in monitoring trial progress and conduct. Research users with PHP have helped to interpret the findings and advised on dissemination.

## Results

### Participant recruitment

**Fig. 1:**
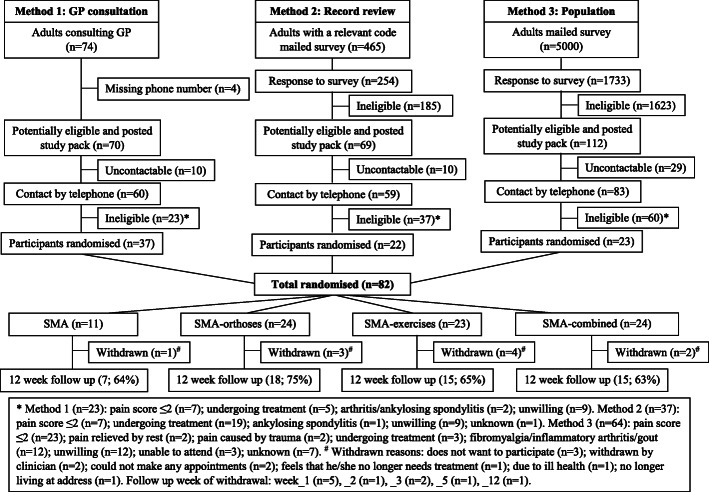
Recruitment, eligibility and retention by participant identification method

Between November 2016 and July 2017, we recruited 82 participants from 251 eligible people invited (median age 55 years (IQR 48, 67), 44% male), giving a recruitment uptake of 32.7% (95%, 90% and 80% lower 1-sided CI_LL_s of 26.9%, 27.8% and 28.8%, respectively). Age and sex of participants and non-participants (*n* = 169) were similar (55.8 vs. 54.8 years; female 56.1% vs. 61.5%). All three identification methods recruited their target number of participants: GP consultation 37/70 potentially eligible (52.9%; 80% 1-sided CI_LL_ 44.5%); MRR 22/69 (31.9%; 24.5%); and population survey 23/112 (20.5%; 15.6%) (Fig. 1). Estimated recruitment rates per year from each identification method were: GP consultation 6.0/10,000 (80%CI 4.8, 7.5) [0.5 per month], MRR 2.6/10,000 (1.9, 3.4) [0.2 per month] and population survey 27.1/10,000 (20.1, 35.8) [at least 2.3 per month]. The mean cost of each identification method per recruited participant was £63.84 for GP consultation, £165.22 for MRR and £1063 for the population survey. Withdrawal rates were 13.5% (5/37), 4.5% (1/22) and 17.4% (4/23) for GP consultation, MRR and the population survey, respectively. Participants recruited by the population survey were older, had milder symptom severity and were less commonly female or employed, whereas those recruited via GP consultation had heel pain of shorter duration and less commonly had bilateral heel pain (Table 1).

**Table 1:** Participant baseline characteristics by intervention arm and by recruitment method

Key characteristics	Total sample *n* = 82	Treatment groups				Recruitment method		
		SMA *n* = 11	SMA-exercises *n* = 23	SMA-orthoses *n* = 24	SMA-combined *n* = 24	GP consultation *n* = 37	Medical record review *n* = 22	Population survey *n* = 23
Non-clinical characteristics								
Age^a^	55 (48, 67)^4^	56 (39, 60)	54 (51, 65)	54 (49, 70)^4^	55 (47, 67)	54 (47, 67)^2^	55 (49, 57)^1^	60 (48, 69)^1^
Gender – males^b^	36 (43.9)	5 (45.5)	11 (47.8)	10 (41.7)	10 (41.7)	14 (37.8)	8 (36.4)	14 (60.9)
Employed^b^	47 (58.0)^1^	7 (63.6)	12 (54.6)^1^	14 (58.3)	14 (58.3)	24 (64.9)	12 (57.1)^1^	11 (47.8)
White UK/European ethnicity^b,e^	40 (88.9)	8 (88.9)	11 (91.7)	9 (100)	12 (80.0)	–	21 (95.5)	19 (82.6)
Effect of pain on work performance^a,c^	2 (0, 6)	6 (0, 7)	3 (0, 6)	1 (0, 4)	0 (0, 2)	1 (0, 5)	2 (0, 7)	2 (0, 3)
Time off work past 6 months^b,c^	4 (8.5)	1 (14.3)	1 (8.3)	0 (0.0)	2 (14.3)	3 (12.5)	1 (8.3)	0 (0)
Clinical characteristics								
Average pain NRS (last 7 days)^a^,^f^	6 (4, 7)^2^	6 (4, 7)	5 (4, 6)^1^	7 (5, 8)	6 (5, 7)^1^	7 (6, 8)	6 (4, 7)	5 (4, 6)^2^
Pain on getting out of bed^a^	7 (5, 9)^2^	7 (5, 8)	7 (5, 8)^1^	8 (7, 10)	7 (5, 8)^1^	8 (7, 9)	6 (5, 8)	6 (5, 7)^2^
First time heel pain^b^	46 (58.2)^3^	4 (36.4)	15 (65.2)	14 (63.6)^2^	13 (56.5)^1^	26 (72.2)^1^	13 (61.9)^1^	7 (31.8)^1^
Heel pain today^b^	74 (90.2)	9 (81.8)	21 (91.3)	22 (91.7)	22 (91.7)	35 (94.6)	18 (81.8)	21 (91.3)
Heel(s) affected^b^								
Left	23 (28.8)^2^	5 (45.5)	7 (31.8)^1^	7 (30.4)^1^	4 (16.7)	14 (40.0)^2^	2 (9.1)	7 (30.4)
Right	24 (30.0)^2^	1 (9.1)	7 (31.8)^1^	8 (34.8)^1^	8 (33.3)	10 (28.6)^2^	9 (40.9)	5 (21.7)
Both	33 (41.3)^2^	5 (45.5)	8 (36.4)^1^	8 (34.8)^1^	12 (50.0)	11 (31.4)^2^	11 (50.0)	11 (47.8)
Pain duration^b^								
< 3 months	15 (18.8)^2^	1 (9.1)	4 (18.2)^1^	5 (20.8)	5 (21.7)^1^	14 (38.9)^1^	0 (0.0)^1^	1 (4.4)
3–6 months	22 (27.5)^2^	2 (18.2)	8 (36.4)^1^	5 (20.8)	7 (30.4)^1^	13 (36.1)^1^	5 (23.8)^1^	4 (17.4)
6–12 months	13 (16.3)^2^	1 (9.1)	4 (18.2)^1^	5 (20.8)	3 (13.0)^1^	3 (8.3)^1^	4 (19.1)^1^	6 (26.1)
> 12 months	30 (37.5)^2^	7 (63.6)	6 (27.3)^1^	9 (37.5)	8 (34.8)^1^	6 (16.7)^1^	12 (57.1)^1^	12 (52.2)
Ache or pain lasting one day or longer in past month^b^	77 (95.1)^1^	11 (100.0)	21 (95.5)^1^	23 (95.8)	22 (91.7)	34 (91.9)	22 (100)	21 (95.5)^1^
FFI-Pain^a,g^	53 (38, 67)^37d^	58 (44, 69)^5^	41 (36, 51)^7^	62 (38, 67)^11^	56 (53, 61)^14^	62 (41, 72)^17^	54 (34, 59)^6^	40 (34, 45)^14^
FFI-Disability^a^	43 (18, 57)^6^	35 (25, 54)^1^	33 (16, 47)^1^	48 (20, 58)^2^	51 (18, 62)^2^	49 (35, 61)^3^	28 (5, 58)	27 (15, 49)^3^
FFI-Activity Restriction^a^	3 (0, 7)^4^	5 (2, 19)^1^	2 (0, 5)^2^	4 (0, 7)	3 (0, 5)^1^	4 (1, 10)^1^	2 (1, 6)^1^	2 (0, 5)^2^
FFI-Overall^a^	99 (62, 133)^38^	103 (86, 148)^5^	69 (58, 111)^8^	127 (66, 134)^11^	107 (62, 156)^14^	124 (75, 153)^17^	74 (57, 123)^6^	83 (64, 102)^17^
MFPDI-Function^a^	18 (16, 22)^9^	19 (5, 24)^1^	17 (14, 22)^4^	19 (15, 23)^2^	20 (17, 22)^2^	20 (17, 22)^1^	18 (15, 21)^4^	16 (14, 23)^4^
MFPDI-Pain^a,h^	13 (12, 16)^4^	13 (11, 15)^1^	13 (11, 14)^1^	15 (12, 17)^2^	13 (12, 16)	14 (12, 16)^1^	15 (11, 17)^1^	12 (11, 14)^2^
MFPDI-Personal Appearance^a^	3 (2, 4)^30^	4 (3, 5)^3^	4 (3, 4)^9^	3 (3, 4)^11^	3 (2, 3)^7^	3 (3, 4)^14^	3 (2, 3)^7^	3 (2, 4)^9^
MFPDI-Overall^a^	36 (31, 40)^34^	36 (30, 43)^3^	32 (30, 38)^10^	37 (33, 40)^12^	37 (29, 39)^9^	38 (32, 40)^16^	35 (33, 39)^9^	35 (28, 40)^9^
EQ5D^a^	.69 (.55, .74)^4^	.68 (.61, .71)^1^	.74 (.70, .77)^1^	.64 (.46, .74)^2^	.69 (.59, .74)	.65 (.46, .73)^2^	.72 (.65, .80)^1^	.70 (.64, .74)^1^

^a^Summarised as median (interquartile range)

^b^Summarised as number (percentage). ^1–38^ Numbers noted after summary statistics relate to the number of missing/ambiguous data (no number denotes complete 82 responses to that measure). For the multi-item measures item-completion numbers, the count × completed-items summary was as follows: FFI-Pain (1 × 1, 1 × 3, 4 × 6, 29 × 7, 2 × 8, 45 × 9); FFI-Disability (1 × 0, 1 × 4, 2 × 7, 2 × 8, 76 × 9); FFI-Activity Restriction (3 × 0, 1 × 2, 78 × 5); MFPDI-Function (1 × 1, 1 × 4, 1 × 6, 1 × 7, 2 × 8, 3 × 9, 73 × 10); MFPDI-Pain (1 × 0, 1 × 2, 2 × 5, 78 × 7); MFPDI-personal appearance (15 × 0, 15 × 1, 52 × 2)

^c^among those answered ‘employed’

^d^Missing data of FFI-Pain mainly due to non-response of two particular questions ‘When you walk with orthotic devices/insoles’ and ‘When you stand with orthotic devices/insoles’

^e^Ethnicity was assessed in the screening survey but not the baseline questionnaire (and was therefore not available for those identified by GP consultation who completed only the baseline questionnaire). Data were available for 45 participants (SMA 9, SMA-exercises 12, SMA-orthoses 9, SMA-combined 15; GP consultation 0, medical record review 22, population survey 23)

^f^For pain NRS, mean (SD) values were as follows: total sample, mean = 5.8 (SD 2.0; 1-sided 80%CI_UL_ = 2.2; 1-sided 90%CI_UL_ = 2.3; 1-sided 95%CI_UL_ = 2.4); SMA, mean = 5.4 (SD 3.0); SMA-exercises, mean = 5.1 (SD 1.6); SMA-orthoses, mean = 6.5 (SD 2.1); SMA-combined, mean = 6.0 (SD = 1.7)

^g^For FFI-Pain, mean (SD) values were as follows: Total sample, mean = 50.7 (SD 17.3; 1-sided 80%CI_UL_ = 19.1; 1-sided 90%CI_UL_ = 20.1; 1-sided 95%CI_UL_ = 21.0); SMA, mean = 57.0 (SD 13.6); SMA-exercises, mean = 43.7 (SD 13.4); SMA-orthoses, mean = 55.7 (SD 18.9); SMA-combined, mean = 51.5 (SD = 20.7)

^h^For MFPDI-Pain, mean (SD) values were as follows: total sample, mean = 13.7 (SD 2.6; 1-sided 80%CI_UL_ = 2.9; 1-sided 90%CI_UL_ = 3.0; 1-sided 95%CI_UL_ = 3.1); SMA, mean = 13.4 (SD 3.0); SMA-exercises, mean = 13.0 (SD 2.3); SMA-orthoses, mean = 14.5 (SD 2.6); SMA-combined, mean = 13.8 (SD = 2.8)

Recruitment was initially slow. After consultation with the Trial Steering Committee and funder, we halted randomisation to the SMA arm after 11 participants had been allocated to that arm to ensure there were sufficient participants in the three clinician-supported intervention arms to allow intervention fidelity to be assessed fully. However, by increasing the pooled practice population from which participants were identified from 60,000 predicted to 86,000 (12 general practices), we were able to recruit our target sample size of at least 80 participants. In total, 11 participants were randomised to SMA, 23 to SMA-exercises, 24 to SMA-orthoses and 24 to SMA-combined (Fig. 1). Baseline characteristics between arms were similar, although those randomised to SMA had longer duration heel pain and were more commonly employed and reported a previous episode of heel pain. The SMA-orthoses arm had higher mean baseline FFI pain score and both the SMA-orthoses and SMA-exercises arms less commonly had bilateral heel pain (Table 1).

### Intervention adherence, fidelity, satisfaction and credibility

Sixty-one participants in the three clinician-supported intervention arms received their allocated treatment (85.9%; 90% 1-sided lower CI_LL_ 80.6%). They attended a mean of 2.3 consultations (SMA-exercises = 2.3, SMA-orthoses = 1.9, SMA-combined = 2.8). Trial clinicians (7 physiotherapists, 5 podiatrists) successfully delivered interventions in routine clinical appointments (Table 2). Participating clinicians found the exercise and orthosis prescription options sufficiently flexible; however, some physiotherapists lacked confidence adjusting orthoses and felt that additional training would be beneficial. Some clinicians felt that balance and eccentric calf exercise options could have been helpful and the ability to provide participants with more than one pair of their prescribed orthotic device may improve adherence, particularly as seasonal changes in footwear may impact adherence.

**Table 2:** Attendance, intervention delivery and protocol adherence

	SMA-orthoses (*n* = 22)^a^		SMA-exercises (*n* = 20)^a^		SMA-combined (*n* = 22)^a^	
Attended, *n* (%)	20/22 (91)		20/20 (100)		22/22 (100)	
Did not attend, *n* (%)	2/22 (9)		0/20 (0)		0/22 (0)	
Cancelled, n (%)	0/22 (0)		0/20 (0)		0/22 (0)	
Plantar heel pain confirmed, *n* (%)	18/19 (95)^3^		18/20 (90)		18/18 (100)^4^	
Total appointments attended (mean)	42 (1.91)		46 (2.30)		62 (2.82)	
Total appointments including non-attenders (mean)	43 (1.95)		49 (2.45)		66 (3.00)	
Foot posture index, mean (SD)	Left foot	Right foot	Left foot	Right foot	Left foot	Right foot
Talar head palpation	0.83 (0.51)^2^	0.89 (0.58)^2^	0.89 (0.68)^2^	0.84 (0.60)^1^	0.71 (0.64)^1^	0.52 (0.81)^1^
Curves above and below the lateral malleolus	0.33 (0.84)^2^	0.17 (0.86)^2^	0.39 (0.70)^2^	0.21 (0.42)^1^	0.48 (0.87)^1^	0.52 (0.60)^1^
Inversion/eversion of the calcaneus	0.56 (0.78)^2^	0.61 (0.85)^2^	0.56 (0.51)^2^	0.32 (0.58)^1^	0.38 (0.74)^1^	0.52 (0.51)^1^
Prominence in the region of the talonavicular joint	0.44 (0.62)^2^	0.50 (0.71)^2^	0.50 (0.51)^2^	0.32 (0.48)^1^	0.48 (0.75)^1^	0.57 (0.75)^1^
Congruence of the medial longitudinal arch	0.39 (0.85)^2^	0.22 (0.81)^2^	0.61 (0.50)^2^	0.26 (0.56)^1^	0.57 (0.93)^1^	0.57 (0.75)^1^
Abduction/adduction of forefoot on rearfoot	1.0 (0.77)^2^	0.72 (0.67)^2^	0.79 (0.63)^1^	0.70 (0.66)	0.86 (0.85)^1^	0.71 (0.78)^1^
Total	3.87 (2.36)^5^	3.20 (2.93)^5^	3.5 (2.3)^3^	2.6 (1.9)^2^	3.5 (4.0)^2^	3.3 (3.4)^2^
Received allocated treatment, n (%)	21/22 (95)		19/20 (95)		21/22 (95)	
Nominated left foot as trial foot, n (%)	8/19 (42)^3^		12/20 (60)		8/22 (36)	
Orthoses						
Orthotic device received(Vectorthotic, Salfordinsole Firm, or Salfordinsole Flex)	15, 3, 1^3^		–		15, 5, 1^1^	
Additional modification to orthoses, n (%)	10/19 (53)^3^		–		10/21 (48)^1^	
Participant fitted orthoses correctly, n (%)	18/19 (95)^3^		–		20/21 (95)^1^	
Discussed orthoses care instructions with participant, n (%)	19/19 (100)^3^		–		21/21 (100)^1^	
Exercise						
Adequate exercise technique, n (%)	–		17/17 (100)^3^		22/22 (100)	
Home exercise leaflet provided, n (%)	–		16/17 (94)^3^		22/22 (100)	
Sum cost (£) of AHP visits [mean scheduled visits x cost per visit] (mean cost per patient)^b^	£1155.84 (£52.54)		£1317.12 (£65.86)		£1774.08 (£80.64)	
Sum cost (£) of insoles [no. receiving insoles x cost of insoles]^c^	£273		–		£273	
Grand Total intervention GBP cost (mean cost per patient)	£1428.84 (£64.95)^b^		£1317.12 (£65.86)^b^		£2047.08 (£93.05)^b^	

^a^Number accounting for early withdrawals: orthosis *n* = 2, exercise *n* = 3, combined *n* = 2. ^1–5^ Numbers noted after summary statistics relate to the number of missing/ambiguous data (no number denotes complete responses to that measure)

^b^Unit cost for Allied health professionals costed at band 6, £26.88 for 30–45-min consultation (£43 per hour [37])

^c^Mean intervention cost based on mean cost of physiotherapist/podiatrist visits plus a typical £13 cost for pre-fabricated orthotic insoles for the proportion of patients in the orthotic and combined intervention groups that received the insoles

More participants in the SMA-combined (exercise: 13/14, 91%; orthosis: 13/15, 87%) and SMA-orthoses (12/17, 71%) arms agreed/strongly agreed they had adopted advice given than in the SMA control (3/6, 50%) or SMA plus exercise (6/13, 46%) arms. Response to weekly self-report diaries ranged from 42 to 63% across the three clinician-supported intervention arms (Table 3). Self-reported adherence was highest in the SMA-combined arm (range over 12 weeks: SMA-exercises 40–83%, SMA-orthoses 57–87%, SMA-combined 64–100%). ‘Lack of time’ or ‘forgetting’ were the main reasons given for lower adherence in the SMA-exercises arm, although adherence to exercise in the SMA-combined arm was high (80–100%). Participant satisfaction with care received, results of treatment/information received and perception of intervention credibility were higher in the three clinician-supported intervention arms than the SMA arm (Table 4).

**Table 3:** Self-reported adherence in the SMA-exercises, SMA-orthoses and SMA-combined arms

Week	Exercise adherence				Orthosis adherence			
	Exercise as often as prescribed over the last week^b^	Exercise, days over the week	Exercise, times per day over the week	Reasons (where specified) for not completing exercise as prescribed^a^	Wearing insoles as often as prescribed over the last week^b^	Wearing insoles, days over the week	Wearing insoles, hours per day over the week	Reasons (where specified) for not wearing orthoses as prescribed^a^
	SMA-exercises				SMA-orthoses			
1	10/12 (83%)	7 (6, 7)	2 (2, 3)	b^(*n* = 2)^	12/15 (80%)	5 (4, 7)	3 (2, 4)	b^(*n* = 1)^ c^(*n* = 1)^ d^(*n* = 1)^
2	9/12 (75%)	7 (6, 7)	2 (2, 3)	b^(*n* = 3)^ c^(*n* = 1)^	13/15 (87%)	6 (4, 7)	4 (2, 4)	c^(*n* = 1)^
3	7/12 (58%)	7 (4, 7)	2 (1, 3)	a^(*n* = 1)^ b^(*n* = 2)^ c^(*n* = 2)^	10/14 (71%)	5 (2, 7)	4 (2, 4)	a^(*n* = 1)^ b^(*n* = 1)^ d^(*n* = 1)^
4	5/11 (45%)	6 (5, 7)	2 (1, 3)	b^(*n* = 4)^ c^(*n* = 1)^ d^(*n* = 1)^	11/15 (73%)	6 (4, 7)	4 (3, 4)	c^(*n* = 1)^ d^(*n* = 2)^
5	5/11 (45%)	5 (3, 7)	1 (1, 3)	b^(*n* = 6)^	10/14 (71%)	6 (2, 7)	4 (4, 4)	b^(*n* = 1)^ c^(*n* = 1)^ d^(*n* = 1)^
6	7/11 (64%)	7 (4, 7)	2 (1, 3)	b^(*n* = 3)^ d^(*n* = 1)^	9/15 (60%)	6 (1, 7)	4 (3, 4)	b^(*n* = 1)^ c^(*n* = 1)^
7	8/11 (73%)	7 (4, 7)	2 (1, 3)	b^(*n* = 2)^ c^(*n* = 1)^	8/14 (57%)	6 (2, 7)	4 (3, 4)	d^(*n* = 1)^
8	7/11 (64%)	7 (6, 7)	2 (1, 3)	b^(*n* = 3)^ c^(*n* = 1)^	10/14 (71%)	5 (4, 7)	4 (2, 4)	c^(*n* = 1)^ d^(*n* = 2)^
9	6/11 (55%)	6 (4, 7)	2 (1, 2)	a^(*n* = 2)^ b^(*n* = 2)^ d^(*n* = 2)^	9/13 (69%)	5 (2, 7)	4 (2, 4)	a^(*n* = 1)^ c^(*n* = 1)^
10	8/11 (73%)	6 (5, 7)	2 (1, 3)	a^(*n* = 1)^, b^(*n* = 3)^	11/14 (79%)	6 (2, 7)	4 (2, 4)	a^(*n* = 1)^ c^(*n* = 1)^
11	5/10 (50%)	7 (4, 7)	2 (1, 3)	a^(*n* = 2)^ b^(*n* = 5)^	9/13 (69%)	6 (3, 7)	4 (4, 4)	b^(*n* = 1)^ c^(*n* = 1)^ d^(*n* = 1)^
12	4/10 (40%)	5 (2, 6)	2 (1, 3)	a^(*n* = 1)^ b^(*n* = 4)^ d^(*n* = 2)^	8/14 (57%)	6 (2, 7)	4 (4, 4)	a^(*n* = 2)^ d^(*n* = 1)^
	SMA-combined							
1	10/11 (91%)	7 (5, 7)	2 (2, 2)	b^(*n* = 1)^ c^(*n* = 1)^	10/11 (91%)	7 (5, 7)	4 (3, 4)	Missing
2	10/11 (91%)	6 (5, 7)	2 (2, 3)	d^(*n* = 1)^	10/11 (91%)	7 (5, 7)	4 (3, 4)	c^(*n* = 1)^
3	11/11 (100%)	7 (5, 7)	2 (2, 2)	n/a	11/11 (100%)	7 (6, 7)	4 (3, 4)	n/a
4	10/11 (91%)	6 (5, 7)	2 (2, 2)	d^(*n* = 1)^	8/11 (73%)	7 (5, 7)	3 (2, 4)	Missing
5	9/11 (82%)	5 (4, 7)	2 (1, 3)	d^(*n* = 1)^	10/11 (91%)	7 (5, 7)	4 (2, 4)	c^(*n* = 1)^
6	9/10 (90%)	5 (4, 7)	2 (1, 2)	d^(*n* = 1)^	7/10 (70%)	5 (2, 7)	4 (2, 4)	c^(*n* = 2)^ d^(*n* = 1)^
7	10/12 (83%)	5 (5, 7)	2 (2, 2)	b^(*n* = 1)^ d^(*n* = 2)^	9/12 (75%)	6 (4, 7)	4 (2, 4)	c^(*n* = 2)^ d^(*n* = 1)^
8	11/12 (92%)	6 (4, 7)	2 (2, 2)	b^(*n* = 1)^ d^(*n* =1)^	10/12 (83%)	6 (4, 7)	4 (3, 4)	c^(*n* = 2)^
9	11/12 (92%)	7 (5, 7)	2 (2, 2)	b^(*n* = 1)^	9/12 (75%)	6 (4, 7)	4 (2, 4)	c^(*n* = 2)^
10	9/11 (82%)	5 (3, 7)	2 (2, 2)	b^(*n* = 2)^	8/11 (73%)	5 (2, 7)	4 (2, 4)	c^(*n* = 1)^
11	9/11 (82%)	6 (4, 7)	2 (2, 2)	b^(*n* = 1)^ d^(*n* = 1)^	7/11 (64%)	5 (2, 7)	3 (2, 4)	c^(*n* = 1)^
12	8/10 (80%)	6 (4, 7)	2 (2, 2)	b^(*n* = 1)^ d^(*n* = 2)^	7/10 (70%)	6 (3, 7)	3 (2, 4)	Missing

Numbers are frequency counts n/n (percent); median (IQR)

^a^Summary excluded those who answered ‘Yes’ to ‘exercise (or, wearing insoles), completed as prescribed over the week’; proportions of the following reasons: a. because my symptoms were better; b. I forgot/lack of time; c. because the exercises (orthotics) were too difficult (uncomfortable)/made symptoms worse; and d. other

^b^‘Strongly agree’ and ‘Agree’ as yes; ‘Not sure’, ‘Disagree’ and ‘Strongly disagree’ as no. *NA* not applicable, *SMA* self-management advice

**Table 4:** Participant satisfaction and perceived credibility with treatment

	SMA	SMA-exercises	SMA-orthoses	SMA-combined
Satisfaction (1): ‘How satisfied are you with the care received?’				
Very satisfied	1 (14.3)	6 (40.0)	10 (55.6)	10 (66.7)
Quite satisfied	2 (28.6)	5 (33.3)	4 (22.2)	4 (26.7)
No opinion	1 (14.3)	3 (20.0)	3 (16.7)	0 (0.0)
Not very satisfied	1 (14.3)	0 (0.0)	1 (5.6)	0 (0.0)
Not at all satisfied	2 (28.6)	1 (6.7)	0 (0.0)	1 (6.7)
Satisfaction (2): ‘How satisfied are you with the results of treatment?’				
Very satisfied	0 (0.0)	2 (13.3)	5 (27.8)	8 (53.3)
Quite satisfied	2 (28.6)	9 (60.0)	8 (44.4)	3 (20.0)
No opinion	2 (28.6)	2 (13.3)	2 (11.1)	1 (6.7)
Not very satisfied	1 (14.3)	1 (6.7)	3 (16.7)	1 (6.7)
Not at all satisfied	2 (28.6)	1 (6.7)	0 (0.0)	2 (13.3)
Satisfaction (3): ‘How satisfied are you with the information received concerning heel problem?’				
Very satisfied	1 (14.3)	7 (46.7)	7 (38.9)	8 (53.3)
Quite satisfied	3 (42.9)	6 (40.0)	9 (50.0)	5 (33.3)
No opinion	1 (14.3)	2 (13.3)	2 (11.1)	1 (6.7)
Not very satisfied	0 (0.0)	0 (0.0)	0 (0.0)	1 (6.7)
Not at all satisfied	2 (28.6)	0 (0.0)	0 (0.0)	0 (0.0)
Credibility (1): ‘Information booklet or treatment has helped heel pain’				
Very confident	0 (0.0)	2 (15.4)	7 (41.2)	6 (42.9)^1^ / 7 (46.7)^2^
Quite confident	2 (28.6)	8 (61.5)	6 (35.3)	6 (42.9) / 3 (20.0)
Neither	3 (42.9)	2 (15.4)	2 (11.8)	0 (0.0) / 3 (20.0)
Not very confident	0 (0.0)	0 (0.0)	0 (0.0)	1 (7.1) / 0 (0.0)
Not at all confident	2 (28.6)	1 (7.7)	2 (11.8)	1 (7.1) / 2 (13.3)
Credibility (2): ‘Recommending the information or treatment to others’				
Very confident	1 (14.3)	3 (23.1)	8 (47.1)	8 (57.1)^1^ / 7 (46.7)^2^
Quite confident	2 (28.6)	8 (61.5)	6 (35.3)	4 (28.6) / 3 (20.0)
Neither	1 (14.3)	1 (7.7)	1 (5.9)	0 (0.0) / 2 (13.3)
Not very confident	1 (14.3)	0 (0.0)	1 (5.9)	1 (7.1) / 1 (6.7)
Not at all confident	2 (28.6)	1 (7.7)	1 (5.9)	1 (7.1) / 2 (13.3)
Credibility (3): ‘Did the information in booklet or treatment seem to make sense to you?’				
Very Sensible	3 (42.9)	5 (38.5)	6 (35.3)	7 (50.0)^1^ / 8 (53.3)^2^
Quite Sensible	2 (28.6)	6 (46.2)	8 (47.1)	6 (42.9) / 6 (40.0)
No opinion	2 (28.6)	2 (15.4)	2 (11.8)	0 (0.0) / 0 (0.0)
Not very sensible	0 (0.0)	0 (0.0)	0 (0.0)	1 (7.1) / 0 (0.0)
Not at all sensible	0 (0.0)	0 (0.0)	1 (5.9)	0 (0.0) / 1 (6.7)

Numbers are *n* (%). Frequency counts do not always sum to corresponding group totals due to some missing data. 1 relates to perceived credibility of exercises; 2 relates to perceived credibility of orthotics (within SMA-combined arm)

### Adverse events

There were 57 expected, non-serious adverse events in the SMA-exercises arm, 83 in the SMA-orthoses arm and 77 in the SMA-combined arm. For exercise, counts for SMA-exercises and SMA-combined were new/different foot pain/tiredness/stiffness (*n* = 22, *n* = 5 respectively); cramp or soreness in the feet or calf muscle (7, 6); bruising in the feet or calf muscle (0, 0); soreness in other joints (15, 15); and other (13, 13). For orthoses, counts for SMA-orthoses and SMA-combined were cramp or soreness in the feet or calf muscle (32, 18); bruising/blister/skin irritation in the feet (21, 4); soreness in other joints (26, 13); falls (0, 0); and other (4, 3).

### Follow-up and outcomes

The follow-up rate over 12 weeks was 55/82 (67.1%; 90% and 80% lower 1-sided CI_LL_s of 60.4% and 62.7% respectively), ranging between 63 and 75% across intervention arms (Table 5). Because of a technical fault, only the first 55 participants received all weekly text messages. Of 842 total SMS texts sent, there were 582 (69%) responses. Further, 66/82 (80%) responded to 4 or more messages, 60/82 (73%) to at least 6 messages and 38/82 (46%) to 9 or more messages. Item completion rates for MFPDI-pain (100%) were higher than for FFI-pain (85%).

**Table 5:** Summary of outcome measures and healthcare utilisation at 12 weeks by treatment arm

Key characteristics	Total sample (*n* = 55†)	SMA (*n* = 7)	SMA-exercises (*n* = 15)	SMA-orthoses (*n* = 18)	SMA-combined (*n* = 15)
Clinical characteristics					
Average Pain NRS (last 7 days)^a,c^	3 (2, 4)^1^	5 (3, 7)	3 (2, 4)	3 (2, 4)	2 (1, 4)^1^
Pain on getting out of bed^a^	4 (2, 7)^2^	6 (3, 9)	4 (3, 6)^1^	5 (2, 7)^1^	2 (1, 9)
Heel pain today^b^	36 (65.5)	4 (57.1)	11 (73.3)	13 (72.2)	8 (53.3)
Heel(s) affected^b^					
Left	13 (28.3)^9^	3 (42.9)	3 (25.0)^3^	6 (35.3)^1^	1 (10.0)^5^
Right	18 (39.1)^9^	2 (28.6)	5 (41.7)^3^	6 (35.3)^1^	5 (50.0)^5^
Both	15 (32.6)^9^	2 (28.6)	4 (33.3)^3^	6 (35.3)^1^	4 (40.0)^5^
Ache or pain lasting one day or longer in past month^b^	36 (69.2)^3^	6 (85.7)	11 (78.6)^1^	12 (70.6)^1^	7 (50.0)^1^
FFI-Pain^a^,^d^	28 (15, 50)^14^	38 (31, 74)^1^	24 (16, 48)^7^	36 (20, 51)^4^	18 (6, 24)^2^
FFI-Disability^a^	12 (1, 35)^3^	19 (12, 67)	17 (7, 48)^1^	7 (0, 32)^2^	1 (0, 13)
FFI-Activity Restriction^a^	1 (0, 3)^1^	2 (0, 18)	2 (0, 2)^1^	2 (0, 4)	0 (0, 1)
FFI-Overall^a^	37 (21, 80)^17^	52 (45, 164)^1^	40 (27, 95)^8^	40 (26, 82)^6^	21 (8, 36)^2^
MFPDI-Function^a^	16 (13, 20)	14 (13, 21)	16 (12, 20)	16 (14, 21)	14 (10, 19)
MFPDI-Pain^a,e^	12 (10, 14)	12 (9, 16)	13 (10, 15)	12 (10, 14)	10 (9, 12)
MFPDI-Personal Appearance^a^	3 (2, 4)^19^	2 (2, 3)^1^	3 (2, 4)^5^	4 (3, 4)^6^	2 (2, 3)^7^
MFPDI-Overall^a^	29 (23, 38)^19^	29 (25, 32)^1^	34 (26, 36)^5^	36 (27, 39)^6^	23 (22, 28)7
EuroQol EQ5D^a^	.74 (.65, .84)^4^	.63 (.51, .84)^1^	.74 (.72, .80)	.74 (.64, .84)^2^	.84 (.71, 1.00)^1^
Global change, *n* (%)					
Completely recovered	4 (7)	0 (0)	1 (7)	0 (0)	3 (20)
Much better	19 (35)	0 (0)	4 (27)	9 (50)	6 (40)
Better	20 (36)	4 (57)	8 (53)	6 (33)	2 (13)
No change	9 (16)	2 (29)	2 (13)	3 (17)	2 (13)
Worse	2 (4)	0 (0)	0 (0)	0 (0)	2 (13)
Much worse	1 (2)	1 (14)	0 (0)	0 (0)	0 (0)
Healthcare utilisation (other than study intervention)					
Additional health provider visits					
Attended an NHS/private health care provider for heel pain, n (%) ^#1^	9 (16)	1 (14)	3 (20)	2 (11)	3 (20)
GP (NHS), visit count	7	2	3	0	2
Physiotherapist (NHS/Private), visit count	15/2	1/0	9/0	1/0	4/2
Podiatrist or Chiropodist (NHS/Private), visit count	5/1	0/0	0/1	2/0	3/0
Sub-Cost (GBP) total (overall mean)	£877.24 (£15.95)	£100.88 (£14.41)	£379.80 (£25.32)	£80.64 (£4.48)	£315.92 (£21.06)
Investigations and surgery					
Received an investigation or treatment for heel pain, n (%)	0 (0)	0 (0)	0 (0)	0 (0)	0 (0)
Referred for surgery for heel pain, n (%)	0 (0)	0 (0)	0 (0)	0 (0)	0 (0)
Had surgery for heel pain, n (%)	0 (0)	0 (0)	0 (0)	0 (0)	0 (0)
Had inpatient stay for heel pain, n (%)	0 (0)	0 (0)	0 (0)	0 (0)	0 (0)
Medications—prescriptions					
Prescribed tablet medication for heel pain, *n* (%) # ^#2^	3 (5)	0 (0)	2 (13)	0 (0)	1 (7)
Ibuprofen (Nurofen, Brufen), n	1	0	1	0	0
Codeine, n	1	0	1	0	0
Cocodamol (Solpadol, Kapake), n	1	0	0	0	1
Sub-Cost (GBP) total (overall mean)	£9.78 (£0.18)	£0	£5.81 (£0.39)	£0	£3.97 (£0.26)
Medications–over-the-counter (OTC)					
Bought over-the-counter medication, n (%) ^#3^	15 (27)	4 (57)	3 (20)	5 (28)	3 (20)
Paracetamol, *n*	6 (mean cost, £3.81)	2	1	2	1
Ibuprofen, *n*	9 (mean cost, £6.37)	3	2	3	1
Co-codamol, *n*	1#	0	0	0	1
Creams/gels/sprays, *n*	3 (mean cost, £2.98)	2	0	0	1
Sub-Cost (GBP) total (overall mean)	£93.10 (£1.69)	£32.69(£4.67)	£16.55 (£1.10)	£26.73 (£1.49)	£17.13 (£1.14)
Indirect (lost productivity)					
No. having time off work (absenteeism), n (days) ^#4^	1 (4)	0 (0)	1 (4)	0 (0)	0 (0)
Sub-cost (GBP) total (overall mean)	£375.42 (£6.83)	£0 (£0)	£375.42(£25.03)	£0 (£0)	£0 (£0)
Work performance (presenteeism) score, mean (SD) ^#5^	1.9 (2.6)	2.3# (3.9)	2.1 (2.0)	2.5 (3.0)	0.6 (1.8)
Sub-cost (GBP) total (overall mean)	£5817.16 (£105.77)	£1205.41 (£172.20)	£1678.96 (£111.93)	£1657.43 (£92.08)	£904.06 (£60.27)
Comparison of aggregated mean costs (GBP)					
Direct healthcare cost (NHS + OTC)	£82.42	£19.08	£92.67	£70.92	£115.51
Societal cost					
Healthcare + Work-absenteeism	£89.25	£19.08	£117.70	£70.92	£115.51
Healthcare + absenteeism + presenteeism	£195.02	£191.28	£229.63	£163.00	£175.78

#1 Unit cost sources (per visit): GP (£37), allied health professionals band 6 (£26.88 per 30–45 minute-consultation) (private costed as NHS due to lack of available source data on private unit costs)) [38]. #2 Prescription cost ibuprofen 200mg 48 tabs (£1.84), codeine phosphate/co-codamol 30 mg/100 mg 100 tabs (£3.97) [39] #3 Self-reported OTC costs; ^co-codamol cost was not recorded (hence taken to be the same as prescription cost). #4 one person reported taking time off work for heel pain; occupation aligned to SOC2010 code 8111 indicating mean hourly wage £11.14 (mean weekly hours worked 42.1); hence 33.7 hours absence assumed over 4 days; cost £375.42 [40]. #5 Mean hourly wage across all employees in 2017 was £16.16 and mean hours worked was 33.3. Presenteeism costs were extrapolated: number of baseline employees x (average of baseline and follow-up work performance scores)/10 x 33.3 x 16.16.

^a^Median (interquartile range)

^b^As number (percent) ^1–19^ Number noted after summary statistics relate to the number of missing/ambiguous data (no number denotes complete 55 responses). For the multi-item measures item-completion numbers, the count × completed-items summary were: FFI-Pain (1 × 0, 1 × 1, 1 × 3, 1 × 6, 9 × 7, 1 × 8, 41 × 9); FFI-Disability (1 × 7, 2 × 8, 52 × 9); FFI-Activity Restriction (1 × 4, 54 × 5); MFPDI-Function (55 × 10); MFPDI-Pain (55 × 7); MFPDI-Personal Appearance (8 × 0, 11 × 1, 36 × 2).

^c^For NRS-Pain, mean (SD) values were: SMA, mean = 5.3 (SD 2.5); SMA + E, mean = 2.9 (SD 1.3); SMA + O, mean = 3.2 (SD 2.3); SMA + C, mean = 2.7 (SD = 3.0). The weighted average SD = 2.3 (1-sided 80%CI_UL_ = 2.5; 1-sided 90%CI_UL_ = 2.7; 1-sided 95%CI_UL_ = 2.8)

^d^For FFI-Pain, mean (SD) values were SMA, mean = 48.3 (SD 27.6); SMA-exercises, mean = 29.6 (SD 16.5); SMA-orthoses, mean = 35.0 (SD 21.4); SMA-combined, mean = 25.2 (SD = 27.1). The weighted average SD = 23.5 (1-sided 80%CI_UL_ = 26.2; 1-sided 90%CI_UL_ = 27.6; 1-sided 95%CI_UL_ = 28.9)

^e^For MFPDI-Pain, mean (SD) values were SMA, mean = 12.7 (SD 4.0); SMA-exercises, mean = 12.6 (SD 3.5); SMA-orthoses, mean = 12.5 (SD 2.9); SMA-combined, mean = 11.6 (SD = 4.2). Weighted average SD = 3.6 (1-sided 80%CI_UL_ = 3.9; 1-sided 90%CI_UL_ = 4.1; 1-sided 95%CI_UL_ = 4.3)

All three pain scales were normally distributed and symmetric at baseline with absolute skewness values < 0.5 and similar mean and median values (pain NRS mean = 5.8, median = 6.0; FFI-Pain 50.7, 53.0; MFPDI-Pain 13.7, 13.0). Floor-and-ceiling baseline distributional concerns were small (floor: pain NRS 0-1 recorded by 2 (2.5%), FFI-pain 0-10 by 1 (2.2%) and MFPDI-pain 7-8 by 0 (0%); ceiling: pain NRS 9-10 by 4 (5%), FFI-pain 90-100 and MFPDI-pain 20-21 both 0 (0%)). Responsiveness from baseline to 12 weeks was higher for FFI-pain (0.96) and pain NRS (1.04) than MFPDI-pain (0.57) (Table 6). Correlation with global rating score and EQ5D was higher for FFI-pain change and pain NRS than MFPDI-pain change.

**Table 6:** Responsiveness and association of change in possible primary outcomes with global change and EQ5D

	Baseline mean (SD)	12 weeks mean (SD)	Change^a^, mean (SD)	Responsiveness^b^	With global change		With EQ5D change
					Spearman’s correlation	AUC (95% CI)^c^	Spearman’s correlation
NRS change	5.9 (1.9)	3.3 (2.4)	2.6 (2.5)	1.04	0.60	0.88 (0.79, 0.96)	0.87
FFI-pain change	50.6 (17.7)	32.8 (23.9)	21.6 (22.4)	0.96	0.74	0.84 (0.69, 0.99)	0.71
MFPDI-pain change	14.0 (2.8)	12.3 (3.5)	1.7 (3.0)	0.57	0.57	0.72 (0.58, 0.67)	0.75

^a^Change from baseline to 12 week

^b^Responsiveness statistic (standardised response mean = mean change in score / SD of change scores; usually interpreted against the benchmarks ≤ 0.2: small, 0.5: moderate, ≥ 0.8: large [35])

^c^Global change dichotomised as (completely recovered or much better / somewhat better or no change or somewhat worse or much worse)

**Fig. 2:**
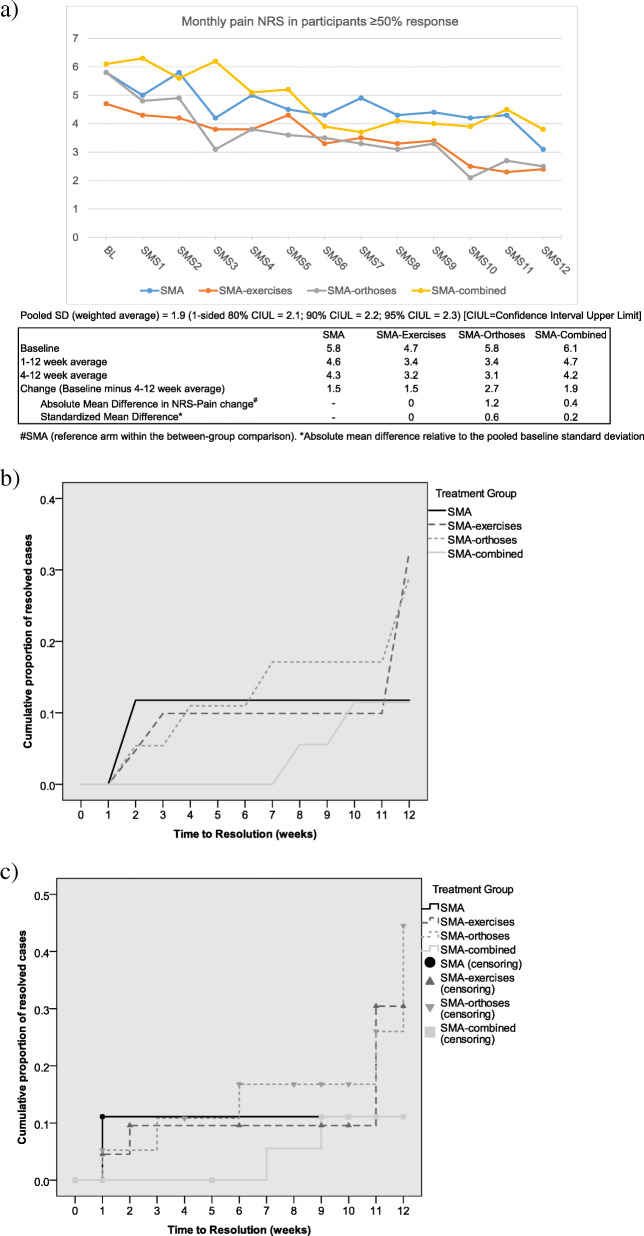
Summary NRS Pain across 12 weeks follow-ups; Kaplan-Meier curves for ‘significantly better’ by treatment arm. **a** Summary mean pain scores. **b** Life-Table graph for SMS pain NRS time-to-event (pain resolution) with straight-line interpolation. **c** Kaplan-Meier graph for SMS pain NRS time-to-event (pain resolution) with stepped-line interpolation

Over 12 weeks, the overall mean reduction in pain NRS was 2.6 (SD 2.5), FFI-pain 21.6 (SD 22.4) and MFPDI-pain 1.7 (SD 3.0). Furthermore, 9/82 (11%) reported being significantly better within the 12-week follow-up (9/55 (16%) of participants who received all weekly text messages) (Table 7). For change in weekly pain NRS, absolute and standardised between-group mean differences scores provided signal of possible effect for the two orthosis arms over the SMA booklet only arm (Fig. 2a). Signal of efficacy was also provided for all three intervention arms versus control (the SMA arm) on pain NRS at 12 weeks: absolute mean difference (standardised difference) 2.1 (1.1) for SMA-exercises, 3.2 (1.7) for SMA-orthoses and 3.2 (1.7) for SMA-combined. For change in FFI-Pain at 12 weeks, the absolute (standardised) mean differences compared to the SMA arm were − 4 (− 0.2) for SMA-exercises, 10 (0.6) for SMA-orthoses and 16 (1.0) for SMA-combined. The median MCID of 1.7 for the pain NRS at 12 weeks was achieved by 34/54 (63%) (SMA 3/7 (43%); SMA-exercises 10/15 (67%), SMA-orthoses 12/18 (67%), SMA-combined 9/14 (64%)). For FFI-pain, the median MCID of 12 was achieved at 12 weeks by 17/28 (61%) (SMA 2/4 (50%), SMA-exercises 4/7 (57%), SMA-orthoses 7/10 (70%), SMA-combined 4/7 (57%)). Pain trajectories were similar across the intervention arms (Fig. 2a).

**Table 7:** Pain NRS by treatment arm (for *n* = 55 participants who received all SMS-text-messages)

Time	All participants			SMA		SMA-exercises		SMA-orthoses		SMA-combined	
	Response (%)	Mean (SD)	Cumulative number significantly better *n* (%)	Mean (SD)	Cumulative number significantly better *n* (%)	Mean (SD)	Cumulative number significantly better *n* (%)	Mean (SD)	Cumulative number significantly better *n* (%)	Mean (SD)	Cumulative number significantly better *n* (%)
BL	55/55(100)	5.6 (1.9)	NA	5.8 (2.1)	NA	4.7 (1.5)	NA	5.8 (2.0)	NA	6.1 (1.6)	NA
W1	40/55 (73)	5.2 (2.3)	NA	5.0 (3.2)	NA	4.3 (2.1)	NA	4.8 (2.1)	NA	6.3 (1.9)	NA
W2	35/55 (64)	5.1 (2.1)	0 (0)	5.8 (2.7)	0 (0)	4.2 (2.2)	0 (0)	4.9 (1.6)	0 (0)	5.6 (2.1)	0 (0)
W3	34/55 (62)	4.4 (2.4)	2 (4)	4.2 (2.4)	0 (0)	3.8 (2.1)	1 (7)	3.1 (2.1)	1 (8)	6.2 (2.2)	0 (0)
W4	40/55 (73)	4.4 (1.9)	3 (6)	5.0 (1.6)	0 (0)	3.8 (2.1)	2 (13)	3.8 (2.0)	1 (8)	5.1 (1.8)	0 (0)
W5	38/55 (69)	4.4 (2.3)	3 (6)	4.5 (2.4)	0 (0)	4.3 (2.5)	2 (13)	3.6 (2.2)	1 (8)	5.2 (2.3)	0 (0)
W6	40/55 (73)	3.7 (2.1)	4 (8)	4.3 (2.7)	0 (0)	3.3 (2.0)	2 (13)	3.5 (2.4)	2 (15)	3.9 (1.7)	0 (0)
W7	37/55 (67)	3.8 (2.4)	6 (12)	4.9 (2.9)	1 (10)	3.5 (1.9)	2 (13)	3.3 (2.8)	3 (23)	3.7 (2.2)	0 (0)
W8	38/55 (69)	3.7 (2.0)	6 (12)	4.3 (2.3)	1 (10)	3.3 (2.0)	2 (13)	3.1 (1.9)	3 (23)	4.1 (2.0)	0 (0)
W9	42/55 (76)	3.7 (2.2)	6 (12)	4.4 (2.0)	1 (10)	3.4 (2.4)	2 (13)	3.3 (1.7)	3 (23)	4.0 (2.4)	0 (0)
W10	40/55 (73)	3.2 (2.4)	7 (14)	4.2 (2.5)	1 (10)	2.5 (1.9)	2 (13)	2.1 (1.6)	3 (23)	3.9 (2.9)	1 (6)
W11	39/55 (71)	3.4 (2.3)	8 (16)	4.3 (1.4)	1 (10)	2.3 (1.8)	2 (13)	2.7 (1.5)	3 (23)	4.5 (3.1)	2 (12)
W12	32/55 (58)	2.9 (2.1)	9 (18)	3.1 (1.9)	1 (10)	2.4 (1.4)	3 (20)	2.5 (1.9)	3 (23)	3.8 (3.1)	2 (12)
FU	36/55 (65)	3.4 (2.5)	NA	5.2 (2.5)	NA	2.7 (1.5)	NA	3.0 (2.1)	NA	3.0 (3.2)	NA

*BL* baseline, *W* week, *FU* follow up questionnaire, *SD* standard deviation, *NA* not applicable. As a result of automated SMS sending errors and host network availability errors, some participants were not sent all messages as described in the protocol. Results were based on 55 participants received all SMS/phone during follow-up (SMA, *n* = 10; SMA-exercises, *n* = 15; SMA-orthoses, *n* = 13; SMA-combined, *n* = 17). ‘Significantly better’ is based on cumulative time taken to consecutive weekly NRS response scores of 0 and/or 1

For informing the sample size for the absolute between-group difference in pain NRS scores, conservative parameter estimates were SD 2.5, baseline-outcome correlation 0.5–0.6, repeated-measures correlation 0.7 and ≥ 80% of participants responding to at least half of text messages received. For the absolute differences in the FFI-pain and MFPDI-Pain, estimates were SD 26 and 4, baseline-outcome correlation 0.4 and < 0.3 and follow-up 50% and 67% respectively.

Baseline variables bilaterally correlated (*r*_s_ ≥ 0.3) with change in pain NRS were pain NRS (0.41) and FFI-Pain (0.31) and with change in FFI-Pain were sex (0.39) and pain NRS (0.33). No baseline variables were correlated with change in MFPDI-Pain. Stepwise (backward/forward) multivariable regression models for change in pain NRS (*p* value cut-off 0.1) included baseline pain NRS, EQ-5D-5L and age (*r* = 0.63). A multiple-correlation coefficient of *r* = 0.63 was obtained from a final stepwise regression model for change in FFI-Pain including baseline FFI-Pain, EQ5D-5L and first-ever episode of heel pain.

The exercise interventions were slightly more expensive from UK-NHS and healthcare perspectives and from a societal perspective including work absenteeism costs only, but costs were broadly comparable across the intervention arms when work presenteeism was taken into account (Table 5).

## Discussion

The TREADON pilot and feasibility trial can inform the design of a future substantive trial of exercise and orthoses for the management of PHP. All three participant identification methods successfully recruited the target number of participants within the required timescale, although a larger target population was required than planned. Baseline characteristics were comparable, although population survey recruits were older and had milder symptoms, and those recruited via GP consultation more commonly had shorter duration and unilateral heel pain. Loss-to-follow-up rates were acceptable but could be improved by offering a wider range of modalities for communication with participants and data collection such as online questionnaires and e-mails in a future main trial.

All interventions were feasible and acceptable to participants and clinicians. Both physiotherapists and podiatrists were able to deliver the interventions to protocol, although some physiotherapists reported lacking confidence in modifying the orthoses and additional training for physiotherapists in the main trial would be necessary. Participants in the three clinician-supported intervention arms attended a mean of 2.3 consultations, whereas the protocol permitted up to 6 sessions. Clinician training in the main trial would need to emphasise the importance of using the full number of available sessions to supervise and progress interventions and we would need to work with participating NHS physiotherapy and podiatry services to ensure this is possible. Participant-reported intervention satisfaction and credibility were high in the three clinician-supported intervention arms but lower in the SMA arm. Signal of efficacy was stronger for SMA-combined and SMA-orthoses than SMA-exercises, although adherence was lower in the SMA-exercises arm. The exercise intervention in a future main trial would need modification to incorporate strategies to enhance adherence. Interestingly, adherence to exercise was better in the SMA-combined arm than the SMA-exercises arm, and the participant burden of receiving the combined intervention did not undermine intervention adherence, satisfaction or credibility. There were no unexpected or serious adverse events.

In terms of outcome measures, the completion rates, responsiveness and correlation with other change scores support using the pain intensity NRS or FFI-pain as the primary outcome in a future main trial. Although MFPDI-pain item completion was high, it is less attractive in view of limited responsiveness which has been reported previously [41, 42]. The pain NRS has the additional advantage of facilitating weekly data collection via SMS text message.

Strengths of the trial include the primary care setting which ensures generalisability to most patients with PHP, and the inclusion of four intervention arms which included a self-management advice control arm and a combined exercises and orthosis arm. The interventions could be delivered by physiotherapists or podiatrists making intervention delivery feasible and scalable for a large future main trial. Limitations include lack of participant blinding, common to many trials of non-pharmacological interventions and collecting only self-reported outcome and adherence data. Because of slow initial recruitment, an amendment to the randomisation ratio was made to ensure sufficient participants were recruited to the clinician-supported interventions to allow adequate assessment of their feasibility. Although the recruitment target was reached, this required a larger pooled practice population from which participants were invited than predicted. The number of participants in each arm was small, particularly in the control arm, limiting the conclusions that can be drawn. The technical issues encountered with the text message follow-ups, whilst restricting the ability to fully assess the pain NRS outcome measure, provided insight into the practical issues concerning this data collection method. Whilst patients were involved in the development of the SMA booklet, the booklet did not include information about the association of PHP with anxiety and depression [2].

## Conclusions

We have demonstrated the feasibility and acceptability of conducting a future, main, randomised clinical trial to investigate the clinical and cost-effectiveness of individualised exercise and/or foot orthoses for PHP. Success criteria concerning recruitment, intervention training and delivery and adherence to, satisfaction with and credibility of interventions were met. Key information to inform participant identification, outcome measures and sample size were obtained. Both physiotherapists and podiatrists delivered both types of intervention successfully, albeit with important learning for clinician training and intervention delivery in the main trial. Descriptive analyses of clinical outcomes provide signal of efficacy and suggest these interventions are promising, and that a future large trial would be informative for clinical practice.

## Supplementary Information

**Additional file 1.** Summary flow of exercise prescription

**Additional file 2.** Protocol for prescription of pre-fabricated foot orthoses

## Data Availability

Keele University is a member of the UK Reproducibility Network and committed to the principles of the UK Concordat on Open Research Data. The School of Medicine and Keele Clinical Trials Unit have a longstanding commitment to sharing data from our studies to improve research reproducibility and to maximise benefits for patients, the wider public, and the health and care system. Metadata, including study protocol, statistical analysis plan, data dictionaries and key study documents (patient information leaflet, blank/coded case report forms, consent form) will be deposited on a publicly accessible repository. De-identified individual participant data (IPD) that underlie the results from this trial will be securely stored on servers approved by a government-backed cyber security scheme and made available to bona-fide researchers upon reasonable request via our controlled access procedures. Unless there are exceptional circumstances, data will be available upon publication of main study findings and with no end date. Data requests and enquiries should be directed to medicine.datasharing@keele.ac.uk. We encourage collaboration with those who collected the data, to recognise and credit their contributions. The data generated from this trial will remain the responsibility of the Sponsor. Release of data will be subject to a data use agreement between the Sponsor and the third party requesting the data. De- identified IPD will be encrypted on transfer.

## Abbreviations

CI: Confidence interval
EQ-5D-5L: EuroQoL
FFI: Foot Function Index
FPI-6: Foot Posture Index-6
GP: General practitioner
IQR: Interquartile range
MCID: Minimal clinically important difference
MFPDI: Manchester Foot Pain and Disability Index
MRR: Medical record review
NRS: Numeric rating scale
PHP: Plantar heel pain
PIL: Participant information leaflet
SD: Standard deviation
SMA: Self-management advice
