# Supplementary material for: Self-management advice, exercise and foot orthoses for plantar heel pain: the TREADON pilot and feasibility randomised trial
Source: Pilot Feasibility Stud. 2021 Apr 1;7:92. doi: 10.1186/s40814-021-00808-0 (PMC8015033; doi:10.1186/s40814-021-00808-0)
Supplement: Supplementary file 1 — Additional file 1. Summary flow of exercise prescription [file 40814_2021_808_MOESM1_ESM.docx]

**Additional file 1**

**Summary flow of exercise prescription**

**Foot/ankle/lower limb assessment**

Individual features targeted as required based on clinical assessment

**Plantar fascia loading**

**Plantar fascia stretching**

**Quadriceps strengthening**

**Quadriceps stretching**

**Calf**

**stretching**

**Hip abduction strengthening**

**Hamstring stretching**

**Intrinsic foot muscle strengthening**

**Lower limb**

**Foot/ankle**

**Stiffness**

**Weakness**

**Weakness**

**Stiffness**

As part of a suite of pre-specific exercises, any combination of the following exercises could be selected, with dose (e.g. number of repetitions, sets and times per day) prescription based on the individual judgement of the treating clinician.

1. None-weight bearing gastrocnemius stretch
2. None-weight bearing soleus stretch
3. Weight bearing gastrocnemius stretch
4. Weight bearing soleus stretch
5. Heel drops from step
6. Plantar fascia stretch (sitting)
7. Plantar fascia stretch (standing)
8. Rolling plantar surface of foot over ball
9. Towel scrunch with toes
10. Pencil pick up with toes
11. Weight bearing unilateral heel raises with toes in dorsiflexion on towel [28]
12. Plantar fascia massage
13. Hip abduction in side lying (Clams)
14. Standing hip abduction
15. Static quadriceps sets
16. Inner range quadriceps
17. Straight leg raise
18. Mini squat
19. None weight bearing hamstring stretch
20. Weight bearing hamstring stretch

Exercise modification was based on a subjective and/or objective re-assessment and included either:

i) progression of prescribed exercises if tolerated with minimal pain and discomfort,

ii) maintenance if tolerated but some moderate pain and discomfort, or

iii) reduction in frequency, duration, intensity or modification of exercise type if not tolerated or adhered to.
