# Supplementary material for: Self-management advice, exercise and foot orthoses for plantar heel pain: the TREADON pilot and feasibility randomised trial
Source: Pilot Feasibility Stud. 2021 Apr 1;7:92. doi: 10.1186/s40814-021-00808-0 (PMC8015033; doi:10.1186/s40814-021-00808-0)

**Additional file 2 - Protocol for prescription of pre-fabricated foot orthoses**

The assessment protocol for the prescription of foot orthoses included two key components to guide the podiatrist/physiotherapist towards the most appropriate foot orthosis prescription.

1. The main driver in influencing the rearfoot posting component of the foot orthosis prescription was the rearfoot posture component of the FPI-6. This was assessed by the podiatrist/physiotherapist with the participant in a relaxed stance position. The podiatrist/physiotherapist observed whether or not the calcaneus was either inverted, vertical, everted, or highly everted (see figure). The remaining factor was assessment of body weight which influenced the selection of the appropriate orthotic device.

2. Each foot orthotic device was fitted according to the size of the participant’s foot using the orthotic device shells of various sizes.

3. Clinicians selected the appropriate first choice device with appropriate rearfoot medial posting dose (figure) in place and assessed the participant for correct size of orthotic device (weight-bearing and non-weight-bearing fit-to-foot).

4. Clinicians then checked the fit of the orthotic device to the shoe (fit-to-shoe).

5. Tolerance was evaluated by asking the participant if they were happy with the comfort and fit of their orthotic device (tolerance). If the participant was not happy with comfort or fit, the clinician could choose to taper the shell density and/or the dose of rearfoot posting.

Foot Orthoses Choice: Our advisory group identified desirable characteristics of an orthotic device intervention protocol as follows:

• an element of patient choice between different device based on comfort and fit

• scope for device adjustment/tailoring by the clinician to provide the desirable level of pronatory control for individual symptoms.

As a result, we developed a pragmatic foot orthosis intervention algorithm which included devices that are prefabricated and modifiable with the use of ‘click-in’ or adhesive additions (medial rearfoot posts) which could be used to change the level of pronatory control, as well as permitting adjustment according to patient comfort and therefore potentially impacting on adherence. Moreover, a range of orthotic shell material densities allowed for a more supportive orthotic device for participants with a higher bodyweight. At present, there is little evidence to suggest that one brand/type of pre-fabricated foot orthoses is more effective than another for the management of PHP.

***Foot Orthoses details***

Vectorthotic devices® (firm density shell), Salfordinsole™ Firm (medium to-firm density shell) and Salfordinsole™ Flex (low-medium density shell)

Materials:.Vectorthotic devices® - semi-rigid polypropylene shell with a closed cell polyethylene cover and polypropylene 2°, 4° and 6° rearfoot posts, Salfordinsole™ shells and 4° rearfoot posts both Sure Step-Control™ thermoplastic elastomer material.

Supplier Details: Participants randomised to receive foot orthoses should receive a trial orthotic device. These were supplied free of charge by Keele CTU in accordance with the Supply Instructions provided in the Investigator Site File.

Storage: Orthotic devices should be stored in accordance with manufacturer’s recommendations and as outlined in the supply instructions provided in the study information.

**Summary Flow chart of foot orthoses prescription**
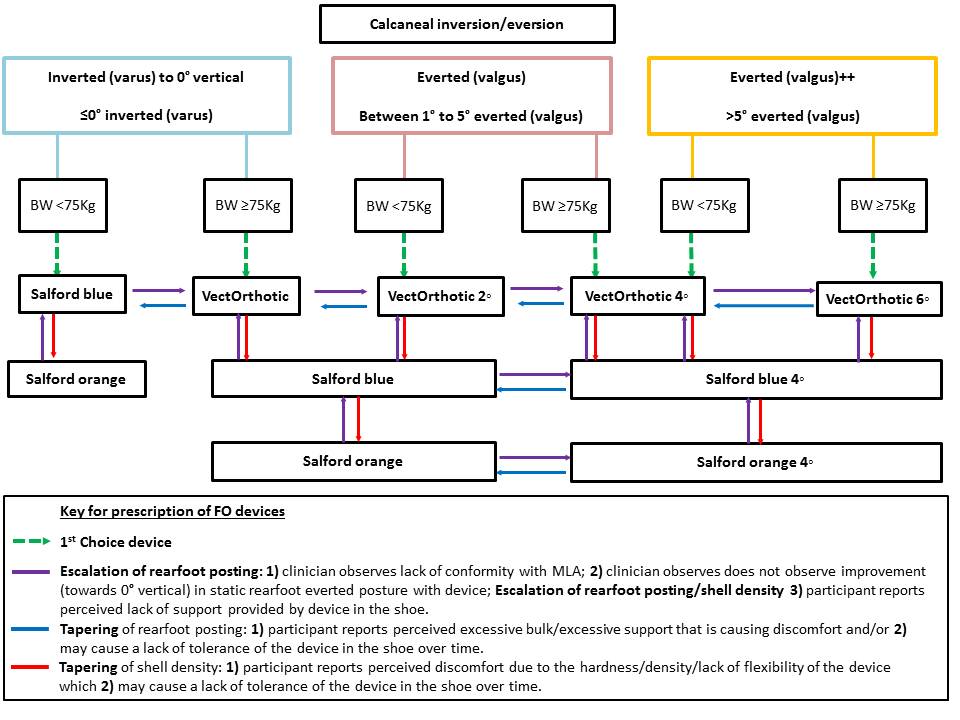

Supplement: Supplementary file 2 — Additional file 2. Protocol for prescription of pre-fabricated foot orthoses [file 40814_2021_808_MOESM2_ESM.docx]
